# A new species of *Buthus* Leach, 1815 from Cyprus (Scorpiones, Buthidae)

**DOI:** 10.3897/zookeys.115.1135

**Published:** 2011-07-05

**Authors:** Ersen Aydın Yağmur, Halil Koç, Wilson R. Lourenço

**Affiliations:** 1 Ege University, Science Faculty, Biology Department, Zoology Section, İzmir, Turkey; 2 Sinop University, Science and Art Faculty, Biology Department, Sinop, Turkey; 3 Muséum national d’Histoire naturelle, Département Systématique et Evolution, UMR7205, CP 053, 57 rue Cuvier 75005 Paris, France

**Keywords:** Scorpion, *Buthus*, new species, Cyprus

## Abstract

During the last decade, several contributions to the genus *Buthus* Leach, 1815 (family Buthidae) and especially to the ‘*Buthus occitanus*’ species complex were proposed. These contributions led to the definition of several species, previously considered only as subspecies or varieties, and also to the description of new species. In the present study, the questionable presence of the genus *Buthus* in the Cyprus is rediscussed and a new species *Buthus kunti* **sp. n.** is described.

## Introduction

The genus *Buthus* was described by Leach, 1815 with the type species (by original designation), *Scorpio occitanus* Amoreux, 1789. The type species was described from Sauvignargues in the South of France. In his study about the scorpions of North Africa, Vachon (see Vachon 1952), revised the composition of the genus *Buthus* and proposed a revised diagnosis, closer to the generic type *Buthus occitanus*. Consequently, quite many species placed in the genus *Buthus* have been transferred to other genera. Some were already available as subgenera, while others have been described by Vachon at this occasion. Can be cited, *Androctonus* Ehrenberg, 1828, *Buthacus* Birula, 1908, *Leiurus* Ehrenberg, 1828, *Compsobuthus* Vachon, 1949 and *Buthotus* Vachon, 1949 (= *Hottentotta* Birula, 1908). (see Lourenço 2002, 2003 for details). However, the classification proposed by Vachon (1952) for the species of *Buthus*, and in particular for those belonging to the “*Buthus occitanus*” species complex, remained unsatisfactory. A more precise definition of the *Buthus* species has been attempted recently by (Lourenço (2002, 2003) which was followed by the elevation of several subspecies to species rank and the description of a new species.

*Buthus occitanus* (Amoreux, 1789) was first recorded from Cyprus by Kraepelin (1891). Levy and Amitai (1980) confirmed it to Cyprus, and also stated that this population was distinct from that of *Buthus israelis* (Shulov and Amitai, 1959), as follows: “Some specimens of *Buthus* from our region resemble specimens of the Moroccan *Buthus occitanus mardochei*”. - “On the other hand, specimens from Cyprus, Tunisia, Libya and Somalia are different”. Subsequently, the presence of a *Buthus* population in the island was again questioned (Gantenbein et al. 2000).

During this study, the third author (WRL) was able to find one adult female previously studied by E. Simon by the end of the 19th century (Simon’s Collection N° 3228) in the collections of the Muséum national d’Histoire naturelle, Paris. In his notes, Simon indicates that the specimen was collected in Cyprus and represented a new species, ‘*Buthus orientalis*’. This species name, however, was never published.

Here we confirm the presence of a *Buthus* population in Cyprus, and a new species belonging to the “*Buthus occitanus*” complex is described. This new *Buthus* population is certainly endemic to Cyprus.

## Materials and methods

Illustrations and measurements were made with the aid of a Wild M5 stereo-microscope with a drawing tube (camera lucida) and an ocular micrometer. Measurements follow Stahnke (1970) and are given in mm. Trichobothrial notations follow Vachon (1974), and morphological terminology mostly follows Vachon (1952) and Hjelle (1990). Specimens were photographed using a Nikon d100 (lens AF micro-NIKKOR 60 mm f/2.8D). Digital images were edited with the assistance of Photoshop CS3 software.

### Abbreviations

MNHNMuseum National d’Histoire Naturelle, Paris, France.

MTASMuseum of Turkish Arachnology Society, Ankara, Turkey.

## Results

### Description of the new species

#### Buthus kunti sp. n.

urn:lsid:zoobank.org:act:96DA8302-0891-4EF8-8D5B-DA8275325908

http://species-id.net/wiki/Buthus_kunti

**Figures 1–3.**
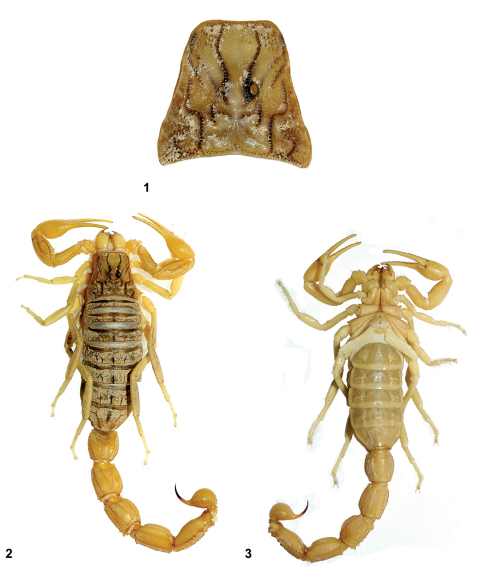
*Buthus kunti* sp. n. **1** Carapace of female holotype **2** female holotype from Karpaz **3** Ditto, ventral view.

Figs 1 [Fig F2] [Fig F3] 12

##### Type material:

Cyprus, 1 female holotype, Karpaz Region, Dipkarpaz Town (İskele), 2 km south east, 35°35'05"N, 34°25'23"E, leg. H. Koç (MTAS). Paratypes: 1 subadult male, Karpaz Region, Zafer headland, 2 km west, 35°41'29"N, 34°33'43"E, leg. M. Z. Yıldız and B. Göçmen (MTAS). 1 subadult male, Güzelyurt District (Morphou), about 5 km south east of Güzelyurt town, leg. H. Koç (MNHN) (Fig. 13).

##### Note:

Although Simon’s female specimen may belong to the new species, we decided not to include it among the type material because (i) it is poorly preserved (ii) the precise collecting site is unknown.

##### Derivatio nominis:

The species is dedicated to Kadir Boğaç Kunt who is the founder of the Turkish Arachnological Society.

##### Diagnosis:

Scorpion of medium to large size, reaching a total length of 73 mm. General coloration yellow to pale yellow, with brownish spots on the carinae of carapace; legs with diffused brownish spots. Carinae moderately to strongly marked; granulations moderately to weakly marked. Fixed and movable fingers with 12 rows of granules. Pectines with 27 to 29 teeth in males, 24–25 in female.

##### Relationships:

*Buthus kunti* sp. n., belongs to the “*Buthus occitanus*” species complex. It can be distinguished from the other species of *Buthus* and in particular from *Buthus israelis* Shulov & Amitai, 1959, a species distributed in the nearby region of the Middle East, by the following characters: (i) *Buthus israelis* is smaller, measuring up to 62 mm in total length for females; (ii) according to Levy and Amitai (1980) pectinal teeth 28–33 in males, 22–28 in females, the new species has a slightly reduced number of pectinal teeth; (iii) metasomal segment II is longer than wide in the female of the new species, whereas it is wider than long in the female of *Buthus israelis*; (iv) pedipalp segments are oligotrichous (sense Vachon 1952) in the new species, whereas they are polytrichous in *Buthus israelis*.

##### Taxonomic note:

As already exposed in a recent paper (Lourenço et al. 2010), the Israeli and Sinai populations were originally described only as a variety: *Buthus occitanus mardochei* var. *israelis* Shulov & Amitai, 1959. Subsequently, this form was raised to subspecies level as *Buthus occitanus israelis* (Levy and Amitai 1980). This decision followed the previous taxonomic position adopted by Vachon (1952), who considered almost all *Buthus* populations from North Africa and Middle East as subspecies of *Buthus occitanus*. However, a revision of the genus *Buthus* (Lourenço 2003) revealed that the species *Buthus occitanus* is limited to France and Spain. Most of the populations of *Buthus*, previously defined as subspecies and even varieties, were raised to the species level, or described as new species. In the case of *Buthus occitanus israelis*, it seemed that this population could no longer be considered as a subspecies of *Buthus occitanus*, both for morphological and especially geographical reasons. Consequently, it was raised to species level, as *Buthus israelis* (Lourenço et al. 2010). Kovařík (2006) examined material from Egypt and Israel and synonimized *Buthus occitanus mardochei* var. *israelis* Shulov & Amitai, 1959 and *Buthus occitanus israelis* with *Buthus intumescens*. But Lourenço et al. (2010) didn’t follow this synonimization and accept *Buthus occitanus israelis* as valid and elevated to species range.

##### Description based on female holotype:

Measurements in Table 1. Coloration basically yellowish to pale yellow (Figs 1–3). Prosoma: carapace yellowish; carinae and eyes marked by dark pigment (Figs 1–2).

**Table 1.** Morphometric values (in mm) of the female holotype of *Buthus kunti* sp. n.

Total length	73.3
Carapace:	
- length	8.2
- anterior width	5.8
- posterior width	9.4
Mesosoma length:	21.4
Metasomal segment I:	
- length	5.5
- width	5.7
Metasomal segment II:	
- length	6.6
- width	5.7
Metasomal segment V:	
- length	8.7
- width	4.8
- depth	3.7
Telson:	
- length	8.0
- width	4.0
- depth	3.5
Pedipalp:	
- Femur length	6.6
- Femur width	2.2
- Patella length	7.6
- Patella width	3.1
- Chela length	13.2
- Chela width	3.4
- Chela depth	3.6
Movable finger: length	8.9

Mesosoma yellowish with carinae also marked by dark pigment, but less conspicuous than carapace. Metasomal segments yellowish; vesicle yellowish; aculeus yellowish at its base and dark reddish at its extremity. Venter yellowish; pectines pale yellow. Chelicerae yellowish with vestigial variegated spots; fingers yellowish with dark reddish to blackish teeth. Pedipalps yellowish; fingers with dark oblique rows of denticles. Legs pale yellow with diffuse brownish spots.

##### Morphology:

Carapace moderately to strongly granular; anterior margin almost straight and without a median concavity. Carinae strong; anterior median, central median and posterior median carinae strongly granular, with ‘lyre’ configuration. All furrows moderate to strong. Median ocular tubercle at the centre of carapace. Eyes separated by almost three ocular diameters (one median eye absent on the holotype). Three pairs of lateral eyes of moderate size (Fig. 1). Sternum triangular, wider than long. Mesosoma: tergites moderately granular. Three longitudinal carinae moderately crenulate in all tergites; lateral carinae reduced in tergites I and II. Tergite VII pentacarinate. Venter: genital operculum divided longitudinally, which plate with a semi-triangular shape. Pectines: pectinal tooth count: 25–24 in female holotype (28–27, 29–29 in male paratypes); middle basal lamella of the pectines not dilated. Sternites without granules, smooth with elongated spiracles; four carinae on sternite VII; other sternites acarinated and with two vestigial furrows. Metasomal segments I to III with ten crenulated carinae, ventral strongly marked on II-III with lobate granules; segment IV with eight carinae, crenulated; the first four segments with a smooth dorsal depression; segment V with five carinae; the latero-ventral carinae crenulate with 2–3 lobate denticles posteriorly (Fig. 5); ventral median carina not divided posteriorly; anal arc composed of 5–6 ventral teeth, and two lateral lobes. Intercarinal spaces weakly granular. Telson almost smooth; aculeus curved and only slightly shorter than the vesicle, without a subaculear tubercle (Fig. 5). Cheliceral dentition as defined by Vachon (1963) for the family Buthidae; external distal and internal distal teeth approximately the same length; basal teeth on movable finger small but not fused (Fig. 7); ventral aspect of both fingers and manus covered with long dense setae. Pedipalps: Femur pentacarinate; patella with eight carinae; all faces weakly granular; chela smooth, without carinae. Fixed and movable fingers with 12 oblique rows of granules. Internal and external accessory granules present, strong; three accessory granules on the distal end of the movable finger next to the terminal denticles (Fig. 6). Legs: Tarsus with two longitudinal rows of thin and long setae ventrally; tibial spur strong on legs III and IV; pedal spurs moderate on legs I to IV. Trichobothriotaxy: trichobothrial pattern of Type A, orthobothriotaxic as defined by Vachon (1974). Dorsal trichobothria of femur arranged in b-configuration (Vachon 1975) (Figs 8–12).

**Figure 4. F2:**
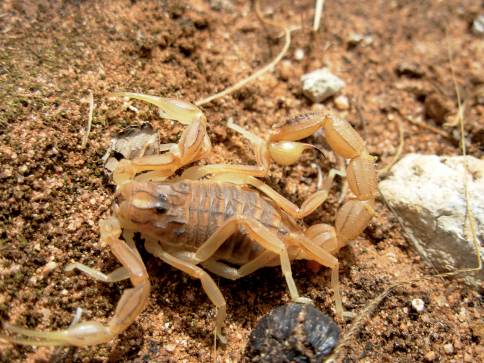
*Buthus kunti* sp. n., subadult male paratype from Zafer headland.

**Figures 5–7. F3:**
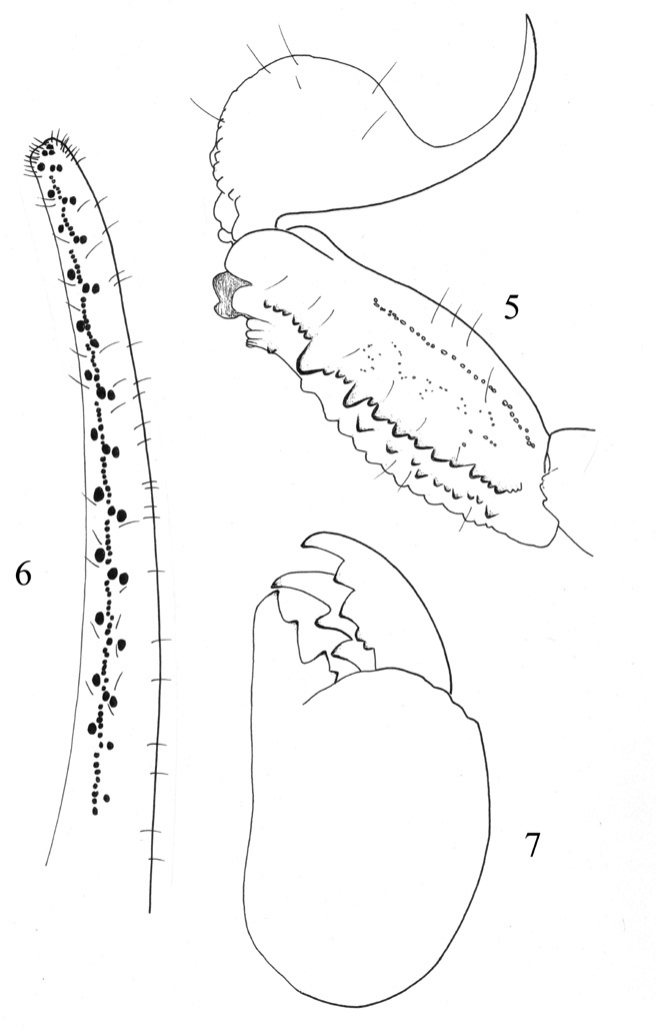
*Buthus kunti* sp. n. Female holotype **5** Metasomal segments V and telson, lateral aspect **6** Movable finger of pedipalp chela with rows of granules **7** Chelicera, dorsal aspect.

**Figures 8–12.**
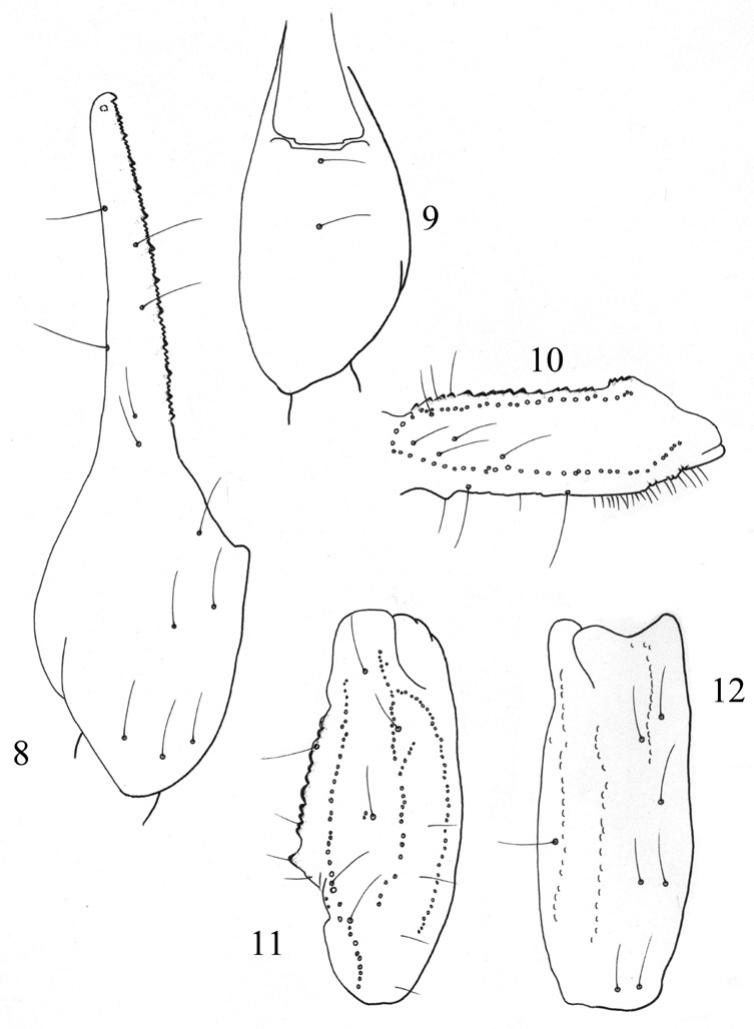
Trichobothrial pattern of *Buthus kunti* sp. n., female holotype. **8–9** Chela, dorso-external and ventral aspects **10** Femur, dorsal aspect **11–12** Patella, dorsal and external aspects.

##### Ecological notes and biogeography:

Cyprus Island exhibits the Mediterranean climate which is warm and rainy in winter and hot and dry in summer. Rainy season is rare and only occurs in winter in plain areas (İlseven et al. 2006). Sandy soil exists at Zafer headland locality, where the vegetation is composed of *Pancratium maritimum*, *Cakile maritima*, *Limonium albidum* and *Pistacia lentiscus* (Fig. 14). Redzina soil is present at Güzelyurt, where the habitat was steppe vegetation with small bushes. *Buthus kunti* sp. n. has allopatric distribution with another species endemic to Cyprus, *Mesobuthus cyprius* Gantenbein & Kropf, 2000. Interestingly, Cyprus Island is the only territory where representatives of *Buthus* and *Mesobuthus* genera have been found together.

**Figure 13.**
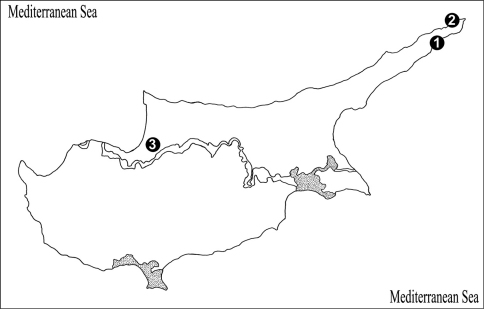
Map of Cyprus, showing the site where the new species was collected. **1** Collecting locality of holotype, Karpaz Region, Dipkarpaz Town (İskele) **2** Collecting locality of paratype, Karpaz Region, Zafer headland **3** Ditto, Güzelyurt District (Morphou).

**Figure 14.**
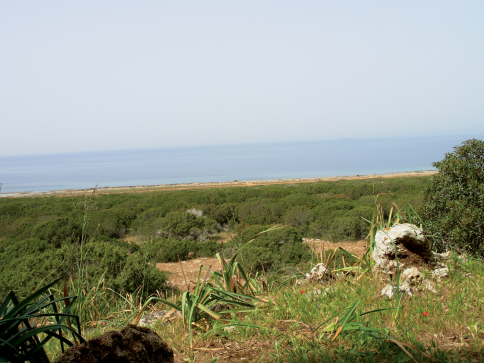
*Buthus kunti* sp. n. Habitat from Zafer headland (Sandy soil habitat).

The geological evolution of the eastern Mediterranean region, has run a series of prominent geological movements, together with the world wide sea levels rising and falling accompanying the continental glaciations leading to join and split of Cyprus and Anatolia (Robertson 1998). It is thus clear that no consensus yet as to the geological history of Cyprus; Schmidt (1960) express Cyprus was part of a united landmass of the mainland and then was broken piece of the mainland, but according to the modern geological history of the eastern Mediterranean region, Cyprus became due to tectonic movements occurring in the area, Gass (1987) supports during Mesozoic time Mt. Troodos is originated a submarine volcano that arise an oceanic island which occured at Cretaceous-Palaeocene. Whereas Kyrenia Mts (which include Pentadactylos Mt.) maybe as a second island or as a part of the southern Taurus Mts range originated in Eocene then later separated from each other to the south (Cavazza and Wezel 2003). According to widely accepted theory is Mediterranean salinity crisis that the Mediterranean sea dried out and these two island or the Trodos island and southern Tauruian-Kyrenian peninsula connected via landbridges about 5.6 Myrs (Hsü et al. 1977; Cavazza and Wezel 2003). When the refilling of the Mediterranean basin, Cyprus terrestrial animals was isolated for around 5.2 – 5.3 Myrs (Robertson 1998; Gantenbein and Keightley 2004 ). This isolation played a major role in forming actual scorpion fauna of Cyprus and molecular and morphological phylogenetic analysis has revealed that populations of the island of Cyprus represent a divergent lineage; so these have been assigned to the species rank (i.e., *Mesobuthus cyprius* Gantenbein and Kropf, 2000). On the other hand, the other discussions about endemism of some snake species occurring in the two island origin of Cyprus (Troodos and Kyrenia island); *Hierophis cypriensis*, in only southern Cyprus (i.e., Throodos island) while *Platyceps najadum* (non-endemic)and *Natrix tessellata* (non-endemic) is distributed only in northern Cyprus (i.e., Kyrenia island) and also on the mainland (Göçmen et al. 2009). Gantenbein and Keightley (2004) stated his analyses shows that *Mesobuthus cyprius* occurring in Cyprus is autochthonous. *Mesobuthus cyprius* recorded in both southern and northern Cyprus. While *Mesobuthus cyprius* recorded at high elevation in Cyprus, *Buthus kunti* sp. n. collected at low altitude in dry condition. It is not yet clear if the distribution of new species restricted to Kyrenia island (northern Cyprus). However, Mt. Troodos run vertically and Kyrenia Mts. lay horizontally with less high in Cyprus, are not usually a zoogeographic barrier there. When we take in consideration for this situation we expect the distribution of new species is all over Cyprus. Another point of view explains that as a result of the geological process, it is a localized endemic species in Kyrenia island (Pentadactylos Mt.).

Since the second record of scorpion species, a museum material, Simon’s material the precise collecting site is unknown and poorly preserved, no other species have been seen in several recent field works, so the species might be very rare on the island, and should be investigated again for male specimens under suitable seasonal conditions.

Unplanned urban settlement destroys the habitats of these endemic species. Government agencies are required to take precautions to not destroy habitats.

## Supplementary Material

XML Treatment for Buthus kunti
